# Motor and neurocognitive profiles of children with symptomatic spinal muscular atrophy type 1 with two copies of *SMN2* before and after treatment: a longitudinal observational study

**DOI:** 10.3389/fneur.2024.1326528

**Published:** 2024-02-21

**Authors:** Ilaria Bitetti, Maria Rosaria Manna, Roberto Stella, Antonio Varone

**Affiliations:** ^1^Pediatric Neurology, Santobono-Pausilipon Children's Hospital, Naples, Italy; ^2^Neurorehabilitation Unit, Santobono-Pausilipon Children's Hospital, Naples, Italy

**Keywords:** gene replacement therapy, motor function, neurocognitive function, onasemnogene abeparvovec, spinal muscular atrophy

## Abstract

**Introduction:**

Spinal muscular atrophy (SMA) is a neurodegenerative disease caused by mutations in the survival motor neuron 1 (*SMN1*) gene. In clinical studies, gene replacement therapy with onasemnogene abeparvovec (formerly AVXS-101, Zolgensma^®^, Novartis) was efficacious in improving motor functioning in children with SMA. However, its effects on cognitive and language skills are largely unknown.

**Methods:**

This longitudinal observational study evaluated changes in motor and neurocognitive functioning over a 1-year period after administration of onasemnogene abeparvovec in 12 symptomatic SMA type 1 patients with two copies of *SMN2* aged 1.7–52.6 months at administration. Motor functioning was measured using the Children's Hospital of Philadelphia Infant Test for Neuromuscular Disorders (CHOP-INTEND) while neurocognitive assessment was measured using Griffiths III. Motor milestones and language ability were also assessed at each timepoint.

**Results and discussion:**

Statistically significant increases in median CHOP-INTEND scores from baseline were observed at 1, 3, 6, and 12 months after onasemnogene abeparvovec administration (all *p* ≤ 0.005). Most (91.7%) patients were able to roll over or sit independently for >1 min at 12 months. Significant increases in the Griffiths III *Foundations of Learning, Language and Communication, Eye and Hand Coordination*, and *Personal-Social-Emotional* subscale scores were observed at 12-months, but not in the *Gross Motor* subscale. Speech and language abilities progressed in most patients. Overall, most patients showed some improvement in cognitive and communication performance after treatment with onasemnogene abeparvovec in addition to significant improvement in motor functioning and motor milestones. Evaluation of neurocognitive function should be considered when assessing the global functioning of patients with SMA.

## 1 Introduction

Spinal muscular atrophy (SMA) is one of the most frequent monogenic neurodegenerative diseases, with an estimated incidence of 1 in 6,000 to 1 in 10,000 live births (1–3). It is divided into four clinical types according to the age at onset and the highest level of function achieved. Type 1 SMA is the most severe; in untreated patients, disease onset occurs before 6 months of age, motor milestones are missed, and death occurs within the first 2 years of life (4).

SMA is caused by mutations in the survival motor neuron 1 (*SMN1*) gene (5) located on the long arm of chromosome 5 in position 5q13. The *SMN2* gene, a paralogue of *SMN1*, produces only 10%−15% of the functional protein expressed by the *SMN1* gene. The disease is characterized by a loss of motor neurons resulting in muscle atrophy and weakness, with the severity dependent on the mutation-related allelic form of *SMN1* and the copy number of *SMN2* (6, 7).

While the clinical phenotype and natural history of SMA are well known in terms of motor, respiratory, and bulbar/swallowing evolution (8–11), there is a paucity of data on the cognitive impact of SMA type 1 in terms of brain, cognitive, and speech/language development (12).

The past decade has seen the development of new therapeutic options, including modulation of *SMN2* splicing and replacement of the *SMN1* gene by gene therapy. Onasemnogene abeparvovec (formerly AVXS-101, Zolgensma^®^, Novartis Gene Therapies EU limited, Dublin, Ireland) is a viral vector-based gene therapy administered via a single intravenous infusion and designed to deliver a functional copy of the human *SMN* gene across the blood-brain barrier. It is authorized in the European Union in patients with SMA 5q with a biallelic mutation in the *SMN1* gene and a clinical diagnosis of SMA type 1, or up to 3 copies of the *SMN2* gene (13) and in the United States in pediatric SMA patients < 2 years of age with biallelic mutations in the *SMN1* gene (14). Clinical studies have demonstrated its efficacy in motor outcomes and favorable safety profile in symptomatic patients with childhood-onset SMA and in presymptomatic infants with two copies of the *SMN2* gene (15–19), supported by real-world data (20–28).

Previously (20), we identified a significant clinical improvement in motor performance over 3 months after gene therapy with onasemnogene abeparvovec in a real-world observational study of symptomatic children with SMA type 1 with two copies of *SMN2* previously treated with nusinersen. With ongoing assessment of this patient population, alongside additional patients, we now describe changes over a 1-year period in their motor performance and neurocognitive profile.

## 2 Materials and methods

Our study cohort included 12 symptomatic patients with SMA type 1 with two copies of *SMN2* who were admitted to the Department of Neurology, AORN Santobono-Pausilipon, Naples, Italy, and treated with onasemnogene abeparvovec between April 2021 and August 2022. Eight of these patients were described in our previous publication (20).

All patients had genetically confirmed SMA 5q with a bi-allelic mutation in the *SMN1* gene and a clinical diagnosis of SMA type 1 or up to 3 copies of the *SMN2* gene, weight < 13.5 kg, and no contraindications to onasemnogene abeparvovec according to the European Medicines Agency (EMA) fact sheet (13). A genetic test, which identified the homozygous deletion of exons 7 and 8 in the *SMN1* gene on chromosome 5q with 95% confidence, confirmed the diagnosis of SMA. There were no specific exclusion criteria.

The local Ethics Committee approved the study and written informed consent was obtained from the parents of all participating patients. Parents were also informed of the risks and benefits of onasemnogene abeparvovec and alternative treatment options, including palliative care.

### 2.1 Therapeutic protocol

A detailed description of the study protocol is available in our previous publication (20). In brief, preliminary investigations were undertaken before administration of onasemnogene abeparvovec as per the EMA product information (13). All patients received a single-dose intravenous infusion of 1.1 × 10^14^ vg/kg onasemnogene abeparvovec over approximately 1 h. Patients also received an immunomodulatory regimen of corticosteroids (13) and underwent motor and respiratory rehabilitation therapy according to the current standard of care. Prior treatment with nusinersen, if used, was at the recommended dosage (29).

Patient management adhered to a multidisciplinary approach and involved neurocognitive assessment, administration of the Children's Hospital of Philadelphia Infant Test for Neuromuscular Disorders (CHOP-INTEND) functional scale, pulmonary assessment, speech therapy, and nutritional and pediatric consultation.

### 2.2 Motor function evaluation

The CHOP-INTEND is a validated scale that reflects measures of disease severity in children with SMA type 1 (30, 31). It is one of the most commonly used outcomes test for non-sitters (32) and has been used in numerous clinical trials as an outcome measure in SMA type 1 (15, 18, 19, 33, 34).

CHOP-INTEND is a reliable measure of motor skills and considers a patient's ability to achieve and sustain specific postures plus the degree of fatigue secondary to respiratory compromise for a maximum score of 64 points. In our study, the CHOP-INTEND was administered to all SMA type 1 patients in clinical practice in a timed manner every 4 months in patients treated with nusinersen, and at baseline (T0) and months 1 (T1), 3 (T3), 6 (T6), and 12 (T12) after intravenous administration of onasemnogene abeparvovec, as requested by the Italian Medicines Agency (AIFA) for drug reimbursement.

In our center, the CHOP-INTEND was administered by a developmental neuropsychomotor therapist and a physiotherapist. Both therapists had undergone specific training for administration of the CHOP-INTEND at the Policlinico Gemelli in Rome (SMA reference center). The CHOP-INTEND was performed using a standardized scale administration procedure. The test was performed approximately 1 h after food intake, on a padded, firm mattress, with the children undressed or wearing only a diaper. The CHOP-INTEND was administered to infants with a behavioral status of 4 or 5 according to the Brazelton Behavioral Rating Scale. The scores were then compared, with the clinical evaluation carried out by the neurologist and confirmed. All patients underwent neuromotor and respiratory therapy on a 5-weekly basis (prescribed at the time of diagnosis).

Motor milestone achievements (head control, rolling, sitting, standing, walking) were also assessed at each timepoint.

### 2.3 Neurocognitive assessments

Neurocognitive assessments were performed by a Pediatric Neuropsychiatrist and a Neurodevelopmental Therapist. Cognitive, language, and motor impairments of all patients were assessed at baseline (T0) and at 6 (T6) and 12 (T12) months after intravenous administration of onasemnogene abeparvovec using the Griffiths III (35). Griffiths III provides an overall measure of a child's development and an individual profile of strengths and needs across five subscales: *Foundations of Learning, Language and Communication, Eye and Hand Coordination, Personal-Social-Emotional*, and *Gross Motor*. The developmental quotient score of each domain was used as the primary outcome measure.

Language ability (vocalizations, babbling, 1–3 words, 10–20 words, sentences) was also assessed at each timepoint.

Additional diagnoses relevant to neurocognitive/behavioral domains (such as intellectual disabilities, autistic spectrum disorder etc.) were not undertaken.

### 2.4 Statistical analysis

Categorical variables are reported as number (percentage), mean ± standard deviation (SD), or median (range). For continuous variables, comparisons between groups were performed using the non-parametric Wilcoxon signed-rank test after running the test for normality (Shapiro-Wilk test) on the whole sample, which rejects the asymmetry hypothesis. *P*-values < 0.05 were considered statistically significant. Analyses were conducted using IBM^®^ SPSS^®^ software.

## 3 Results

### 3.1 Patient population

The patient population consisted of 12 symptomatic children with SMA type 1 with two copies of *SMN2* and a mean age at first treatment of 7.8 ± 6.2 months (range 1.7–25.9 months) (Tables 1, 2). Nine patients had received 4–13 nusinersen administrations prior to study enrollment (mean age 8.7 ± 6.8 months, range 2.0–25.9, at first nusinersen administration), while 3 patients (patients 6, 12, and 13) were nusinersen-naïve. The mean age at onasemnogene abeparvovec administration for all patients was 28.0 ± 20.0 months (range 1.7–52.6 months). None tested positive for COVID-19.

**Table 1:** Demographic and clinical characteristics of all SMA type 1 patients (*n* = 12).

	***n* (%)**
Male	8 (66.7)
Female	4 (33.3)
Age at onset (months)^*^	3.3 ± 1.8 (0–6.0)
Age at diagnosis (months)^*^	7.0 ± 5.7 (2.0–24.1)
Age at first treatment (months)^*^	7.8 ± 6.2 (1.7–25.9)
Previous nusinersen treatment	9 (75.0)
Age at nusinersen administration (months)^*^	8.7 ± 6.8 (2.0–25.9)
Age at onasemnogene abeparvovec administration (months)^*^	28.0 ± 20.0 (1.7–52.6)
PEG	3 (25.0)
NIV	10 (83.3)
Tracheostomy	0 (0)
2 copies of the *SMN2* gene	12 (100)

h, hours; n, number; NIV, non-invasive ventilation; PEG, percutaneous endoscopic gastrostomy; SD, standard deviation. ^*^mean ± SD (range).

**Table 2:** Patient population characteristics.

**Pt**	**Gender**	**Age at onset (mo)**	**Age at diagnosis (mo)**	**Nusinersen**	**Age at nusinersen admin (mo)**	**Age at OA admin (mo)**	**PEG**	**NIV**
Pt 2	F	5.0	6.2	Y	6.6	48.4	N	Y
Pt 3	M	4.0	5.9	Y	6.2	44.4	N	Y
Pt 4	M	4.0	8.9	Y	9.7	45.7	N	Y
Pt 5	M	5.0	6.2	Y	6.7	12.4	N	Y
Pt 6	M	1.0	2.0	N	-	1.7	N	Y
Pt 7	M	2.0	6.4	Y	7.0	11.5	N	Y
Pt 8	M	0.0	2.0	Y	2.0	40.1	Y	Y
Pt 9	F	2.0	5.1	Y	9.3	17.4	Y	Y
Pt 10	M	3.0	4.9	Y	5.0	52.6	Y	Y
Pt 11	M	6.0	24.1	Y	25.9	48.9	N	Y
Pt 12	F	3.0	6.3	N	-	6.8	N	N
Pt 13	F	4.0	6.2	N	-	6.8	N	N

admin, administration; F, female; M, male; mo, months; N, no; NIV, non-invasive ventilation; OA, onasemnogene abeparvovec; PEG, percutaneous endoscopic gastrostomy; Pt, patient; Y, yes.

### 3.2 Motor function

Statistically significant increases in median CHOP-INTEND scores from baseline were observed at all timepoints after onasemnogene abeparvovec administration (T0 to T1, *p* = 0.005; T0 to T3, *p* = 0.003; T0 to T6, *p* = 0.003; T0 to T12, *p* = 0.004) (Table 3).

**Table 3:** CHOP-INTEND total scores at baseline and 12 months after onasemnogene abeparvovec administration in all patients (*n* = 12; mean age 28.0 ± 20.0 months, range 1.7–52.6 months, at onasemnogene abeparvovec administration), patients who received nusinersen prior to study enrollment (*n* = 9; *nusinersen* + *OA*; mean age 35.7 ± 16.9 months, range 11.5 to 52.6 months, at onasemnogene abeparvovec administration), nusinersen-naïve patients (*n* = 3; *OA only*; mean age 5.1 ± 3.0 months, range 1.7 to 6.8 months, at onasemnogene abeparvovec administration), and patients >2 years of age at onasemnogene abeparvovec administration (mean age 46.7 ± 4.3 months, range 40.1 to 52.6 months).

**Patient pop**.	**T0**	**T1**	**T3**	**T6**	**T12**	*p-*value^*^
All patients^†^	41.0 (4–53)	44.5 (14–53)	49.5 (26–58)	51.5 (32–58)	52.5 (36–60)	≤ 0.005
Nusinersen + OA^‡^	42.8 ± 8.9	46.6 ± 6.9	51.0 ± 5.2	52.1 ± 5.6	54.0 ± 4.1	-
OA only^‡^	10.3 ± 5.7	19.3 ± 8.4	32.0 ± 6.0	36.7 ± 7.2	42.7 ± 7.0	-
Patients >2 years^‡^	46.0 ± 5.1	48.5 ± 4.6	52.0 ± 3.8	52.8 ± 4.3	53.7 ± 3.6	-

OA, onasemnogene abeparvovec. T0 = baseline; T1 = 1 month; T3 = 3 months; T6 = 6 months; T12 = 12 months. Results are shown as ^†^median (range) or ^‡^mean ± SD. ^*^Wilcoxon signed-rank test: T0 to T1, p = 0.005; T0 to T3, p = 0.003; T0 to T6, p = 0.003; T0 to T12, p = 0.004.

**Figure 1:**
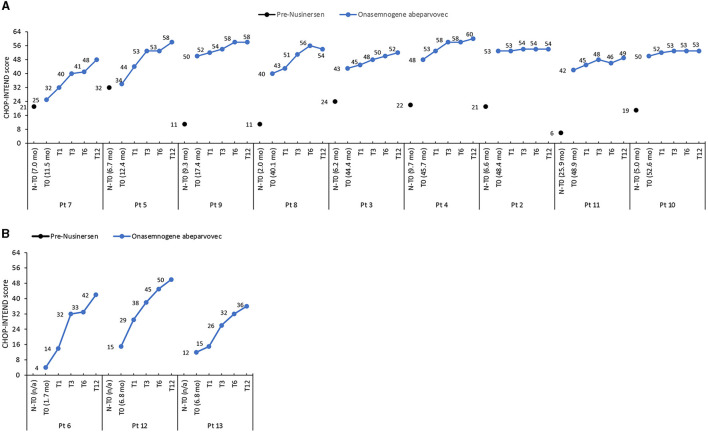
CHOP-INTEND scores in **(A)** patients previously treated with nusinersen (mean age 35.7 ± 16.9 months, range 11.5 to 52.6 months, at onasemnogene abeparvovec administration) and **(B)** nusinersen-naïve patients (mean age 5.1 ± 3.0 months, range 1.7 to 6.8 months, at onasemnogene abeparvovec administration). CHOP-INTEND scores are shown pre-nusinersen (N-T0), if applicable, and at baseline (T0), 1 (T1), 3 (T3), 6 (T6), and 12 (T12) months after intravenous administration of onasemnogene abeparvovec. Patient number and age at first nusinersen administration (N-T0) and at onasemnogene abeparvovec administration (T0) are shown on the horizontal axis.

The mean CHOP-INTEND score increased by 32.4 points from baseline to 12 months in nusinersen-naïve patients (mean age 5.1 ± 3.0 months, range 1.7 to 6.8 months, at onasemnogene abeparvovec administration) and by 11.2 points in patients who had received nusinersen prior to study enrollment (mean age 35.7 ± 16.9 months, range 11.5 to 52.6 months, at onasemnogene abeparvovec administration) (Table 3). All nusinersen-naïve patients had higher CHOP-INTEND scores at 12 months than baseline as did all patients previously treated with nusinersen (Figures 1A, B).

### 3.3 Motor milestones

**Figure 2:**
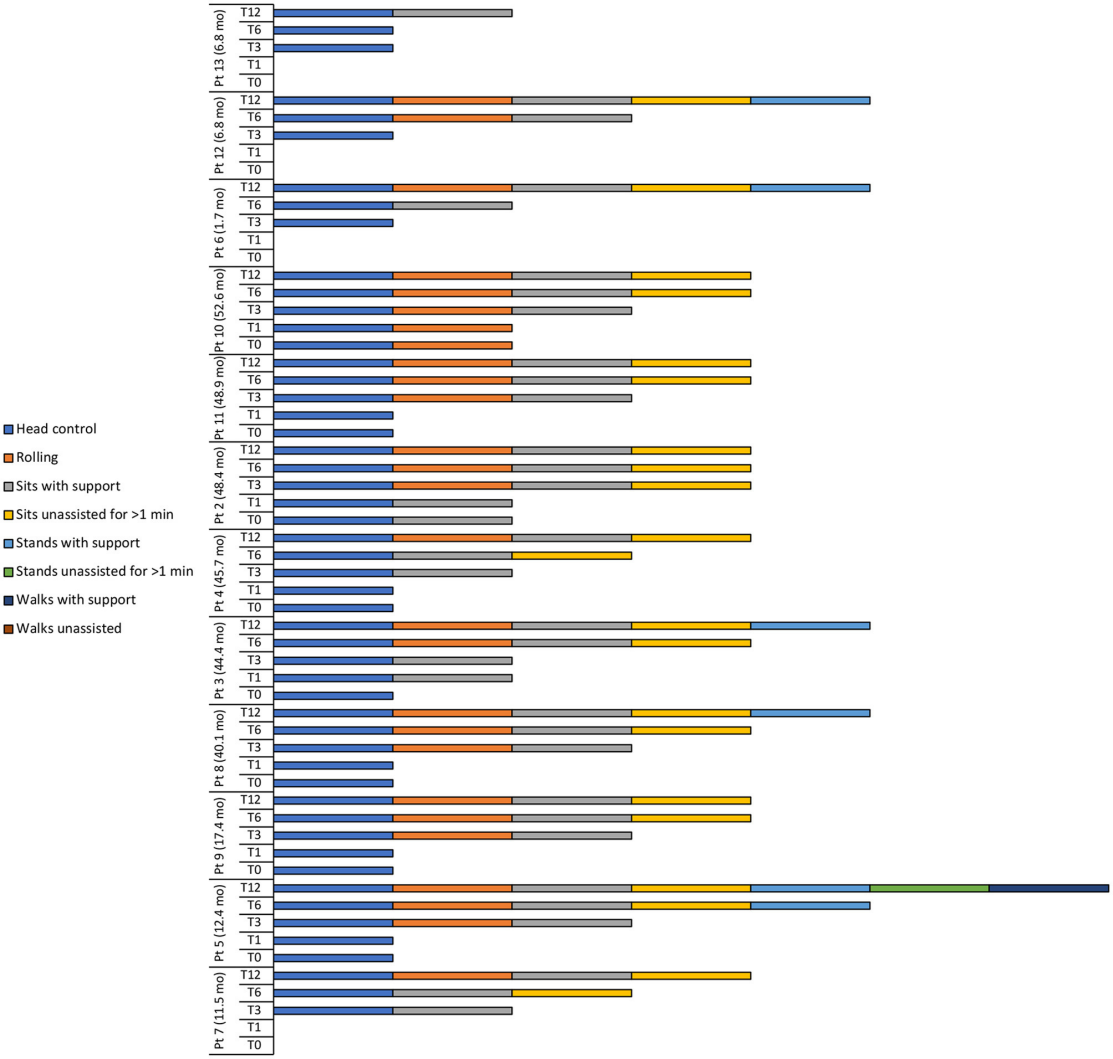
Motor milestone achievements at baseline (T0), 1 (T1), 3 (T3), 6 (T6), and 12 (T12) months after intravenous administration of onasemnogene abeparvovec. Patient number and age at onasemnogene abeparvovec administration are shown on the vertical axis. Patients 6, 12, and 13 were nusinersen-naïve.

At baseline, 8 patients had head control, with patient 10 also able to roll and patient 2 also able to sit with support (Table 4, Figure 2).

**Table 4:** Motor milestone achievements and language ability at baseline and 12 months after onasemnogene abeparvovec administration in all patients (*n* = 12, mean age 28.0 ± 20.0 months, range 1.7–52.6 months, at onasemnogene abeparvovec administration), patients who received nusinersen prior to study enrollment (*n* = 9; *nusinersen* + *OA*; mean age 35.7 ± 16.9 months, range 11.5 to 52.6 months, at onasemnogene abeparvovec administration), and nusinersen-naïve patients (*n* = 3; *OA only*; mean age 5.1 ± 3.0 months, range 1.7 to 6.8 months, at onasemnogene abeparvovec administration).

	**All patients**		**Nusinersen** + **OA**		**OA only**	
	**Baseline**	**12 months**	**Baseline**	**12 months**	**Baseline**	**12 months**
**Motor milestone**						
Head control	8 (66.7)	12 (100)	8 (88.9)	9 (100)	0 (0)	3 (100)
Rolling	1 (8.3)	11 (91.7)	1 (11.1)	9 (100)	0 (0)	2 (66.7)
Sits with support	1 (8.3)	12 (100)	1 (11.1)	9 (100)	0 (0)	3 (100)
Sits unassisted for >1 min	0 (0)	11 (91.7)	0 (0)	9 (100)	0 (0)	2 (66.7)
Stands with support	0 (0)	5 (41.7)	0 (0)	3 (33.3)	0 (0)	2 (66.7)
Stands unassisted for >1 min	0 (0)	1 (8.3)	0 (0)	1 (11.1)	0 (0)	0 (0)
Walks with support	0 (0)	1 (8.3)	0 (0)	1 (11.1)	0 (0)	0 (0)
Walks unassisted	0 (0)	0 (0)	0 (0)	0 (0)	0 (0)	0 (0)
**Language**						
Vocalizations	8 (66.7)	1 (8.3)	5 (55.6)	1 (11.1)	3 (100)	0 (0)
Babbling	1 (8.3)	0 (0)	1 (11.1)	0 (0)	0 (0)	0 (0)
1–3 words	1 (8.3)	3 (25.0)	1 (11.1)	0 (0)	0 (0)	3 (100)
10–20 words	0 (0)	2 (16.7)	0 (0)	2 (22.2)	0 (0)	0 (0)
Sentences	2 (16.7)	6 (50.0)	2 (22.2)	6 (66.7)	0 (0)	0 (0)

OA, onasemnogene abeparvovec. Results are shown as n (%).

At 12 months, all patients achieved head control and were able to sit if supported, while 11 of 12 patients (91.7%) were able to roll or sit unassisted for >1 min (Table 4, Figure 2). Five patients (41.7%) could stand with support, with patient 5 (previously exposed to nusinersen) also able to stand unassisted for >1 min and walk with support.

Two of 3 nusinersen-naïve patients (66.7%) (mean age 5.1 ± 3.0 months, range 1.7 to 6.8 months, at onasemnogene abeparvovec administration) were able to stand with support at 12 months, whereas only 3 of 9 patients (33.3%) who had received nusinersen prior to study enrollment (mean age 35.7 ± 16.9 months, range 11.5 to 52.6 months, at onasemnogene abeparvovec administration) achieved this milestone (Table 4, Figure 2).

### 3.4 Neurocognitive assessments

Significant increases in the median developmental quotient scores of all patients were identified between baseline (T0) and 6-months (T6) in the Griffiths III *Foundations of Learning* (*p* = 0.041) and *Personal-Social-Emotional* (*p* = 0.028) subscales, but not in the *Language and Communication, Eye and Hand Coordination*, or *Gross Motor* subscales (Table 5). Between baseline (T0) and 12-months (T12), significant increases were identified in the *Foundations of Learning* (*p* = 0.005), *Language and Communication* (*p* = 0.003), *Eye and Hand Coordination* (*p* = 0.004), and *Personal-Social-Emotional* (*p* = 0.006) subscales, but not in the *Gross Motor* subscales (Table 5).

**Table 5:** Motor function and neurocognitive assessments using Griffiths III at baseline and at 6 and 12 months after onasemnogene abeparvovec administration in all patients (*n* = 12).

**Subscale^†^**	**T0**	**T6**	**T12**	***p-*** **value** ^*^		
				**T0–T6**	**T6–T12**	**T0–T12**
Foundations of learning	26 (20–84)	42.5 (20–100)	68.5 (20–102)	**0.041**	**0.003**	**0.005**
Language and communication	46.5 (20–86)	56.5 (20–91)	70 (20–123)	0.123	**0.003**	**0.003**
Eye and hand coordination	34 (20–83)	50.5 (20–90)	68 (20–98)	0.056	**0.005**	**0.004**
Personal-social-emotional	49 (20–84)	61.5 (20–95)	70 (20–92)	**0.028**	**0.011**	**0.006**
Gross motor	20 (20–69)	20 (20–29)	20 (20–20)	0.109	0.655	0.144

OA, onasemnogene abeparvovec. ^†^Developmental quotient scores are shown as median (range). T0 = baseline; T6 = 6 months; T12 = 12 months. ^*^Wilcoxon signed-rank test. Bold values indicate statistically significant.

**Figure 3:**
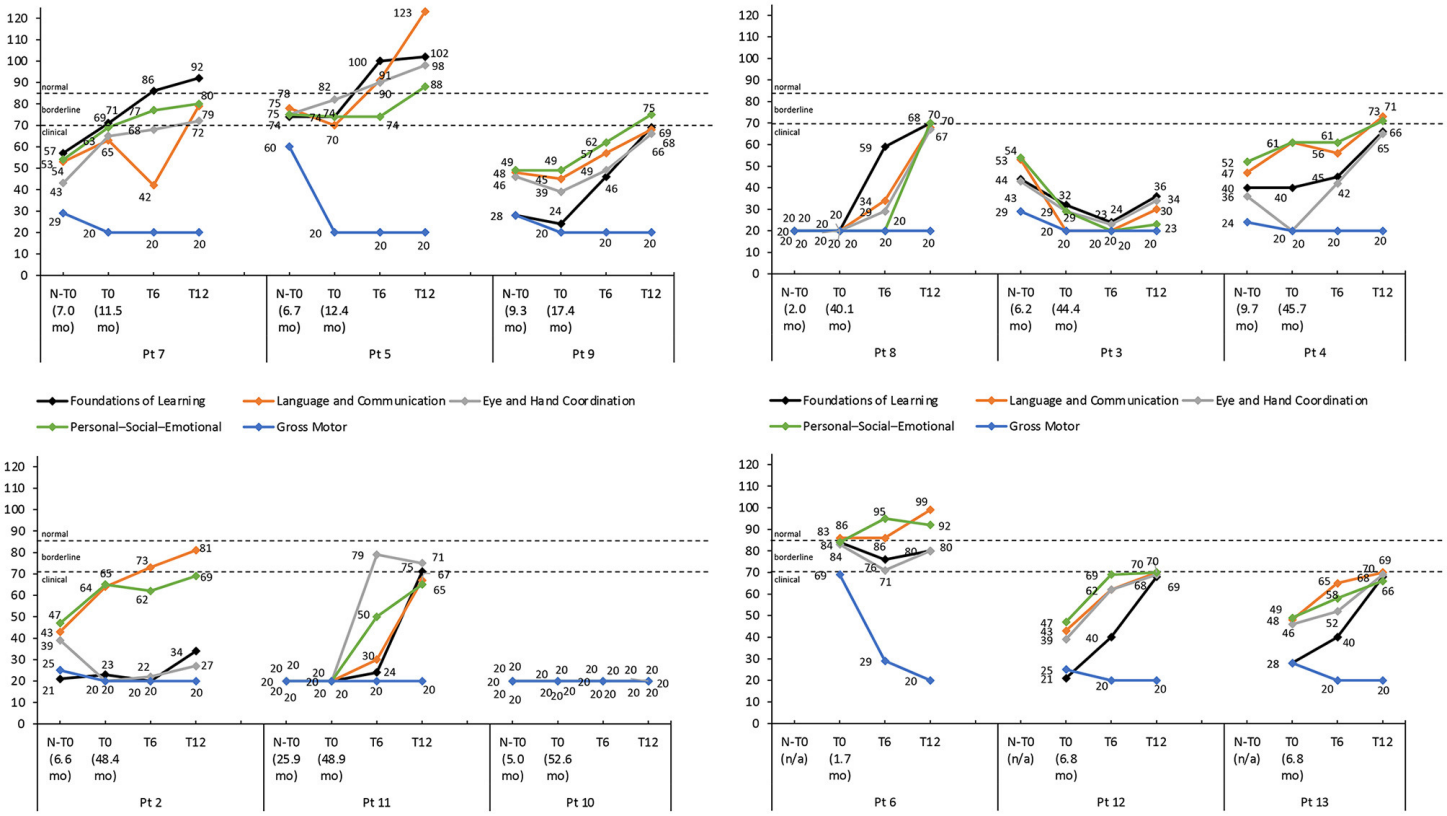
Griffiths III subscale developmental quotient scores for each patient with thresholds of clinical significance (clinical < 70; borderline 70–84; normal >84). Scores are shown pre-nusinersen (N-T0), if applicable, and at baseline (T0), 6 (T6), and 12 months (T12) after intravenous administration of onasemnogene abeparvovec. The horizontal axis shows patient number and age at first nusinersen administration (N-T0) and at onasemnogene abeparvovec administration (T0). Patients 6, 12, and 13 were nusinersen-naïve.

The developmental quotient scores of most Griffiths III subscales, except the *Gross Motor* subscale, increased over the 12-month period after onasemnogene abeparvovec administration regardless of the nusinersen status of the patients (Figure 3). Despite this, 9 patients had low to borderline scores ( ≤ 84) across all subscales, indicative of an overall developmental delay, and only 3 patients (patients 7, 5, and 6) had normal scores (>84) for any of the subscales.

### 3.5 Speech and language development

**Figure 4:**
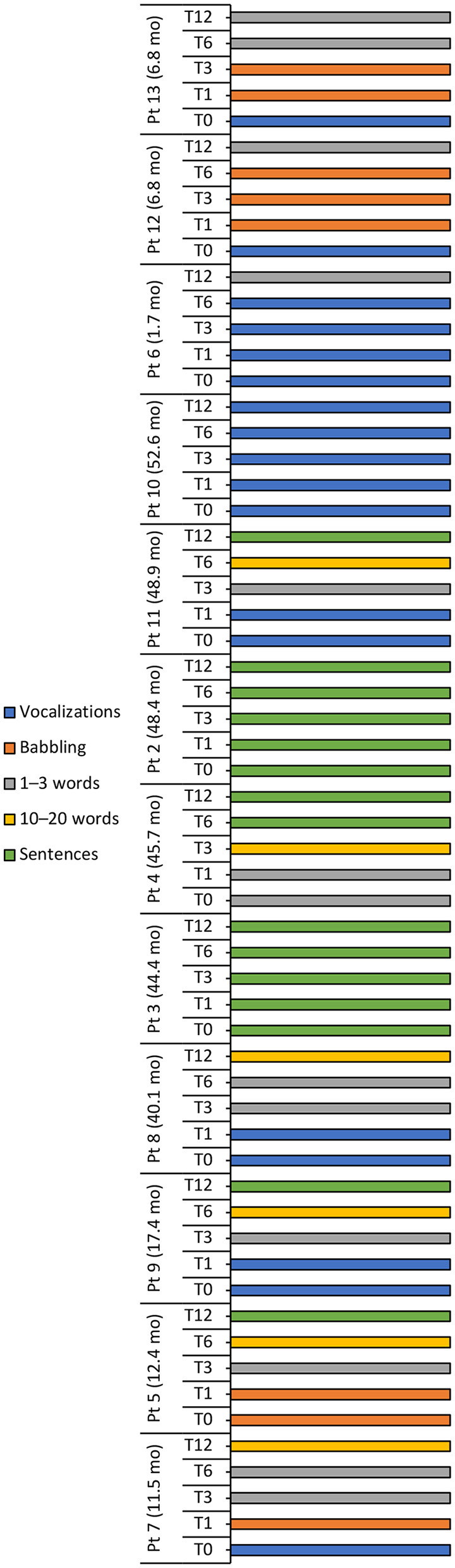
Language milestone achievements at baseline (T0), 1 (T1), 3 (T3), 6 (T6), and 12 (T12) months after intravenous administration of onasemnogene abeparvovec. Patient number and age at onasemnogene abeparvovec administration are shown on the vertical axis. Patients 6, 12, and 13 were nusinersen-naïve.

A positive trend in speech and language development was observed over the 12-month period in most patients, with half the patient population speaking *Sentences* at 12 months, three patients (25%) speaking *1–3 words* and two patients (16.7%) speaking *10–20 words* (Table 4, Figure 4). Conversely, speech development in patient 10 did not progress beyond *Vocalizations*.

The language ability of all three nusinersen-naïve patients (mean age 5.1 ± 3.0 months, range 1.7 to 6.8 months, at onasemnogene abeparvovec administration) improved from *Vocalizations* at baseline to *1–3 words* at 12-months, while eight nusinersen-experienced patients (mean age 35.7 ± 16.9 months, range 11.5 to 52.6 months, at onasemnogene abeparvovec administration) achieved either *10–20 words* or *Sentences* over the 12-month period (Table 4, Figure 4).

### 3.6 Outcomes in children over 2 years of age

Six patients were >2 years of age at onasemnogene abeparvovec administration (mean age 46.7 ± 4.3 months), with age at administration ranging from 40.1 months (patient 8), 44.4 months (patient 3), 45.7 months (patient 4), 48.4 months (patient 2), 48.9 months (patient 11), to 52.6 months (patient 10) (Table 2). All 6 patients had received nusinersen prior to study enrollment.

In these patients, the mean CHOP-INTEND score increased by 7.7 points from baseline to 12 months (Table 3), and scores were higher at 12 months than baseline in all patients, although the increase was marginal in patients 2 and 10 (Figure 1A).

Motor milestone achievements were observed in all patients. At 12 months, patients 4, 2, 11, and 10 could sit unassisted for >1 min, while patients 8 and 3 could stand with support (Figure 2).

The developmental quotient scores of most Griffiths III subscales increased over the 12-month period, except the *Gross Motor* subscale (Figure 3).

An assessment of speech and language development showed that language ability improved in patients 8, 4, and 11, patients 3 and 2 were able to form sentences at baseline and retained this ability over the 12-month period, and patient 10 did not progress beyond *Vocalizations* (Figure 4).

## 4 Discussion

To the author's knowledge, this is the first longitudinal observational study to evaluate the change in motor and neurocognitive functioning over 1 year in 12 symptomatic patients with SMA type 1 with two copies of *SMN2* treated with onasemnogene abeparvovec.

Our results identified significant clinical improvements in motor function performance at all timepoints up to 12-months, as well as the attainment of motor milestones in all patients. Compared with baseline, significant increases in learning and language abilities were also identified at 12 months when assessed using Griffiths III subscales, whereas the *Gross Motor* subscale showed a decline in the developmental progression of gross motor functions. Most patients experienced a positive trend in speech and language development over the 12-month period after onasemnogene abeparvovec administration.

Our clinical findings concur with findings from the clinical registration studies and long-term follow-up (START, STR1VE, and STR1VE-EU) (15–18), highlighting the beneficial sustained clinical durability of onasemnogene abeparvovec over time. Notably, the significant increase from baseline in median CHOP-INTEND scores and achievement of new motor milestones, including walking with support in one patient, underscores the use of onasemnogene abeparvovec for treatment of symptomatic SMA type 1 patients regardless of prior exposure to nusinersen.

An increase in CHOP-INTEND scores has been demonstrated for all three drugs approved by the US Food and Drug Administration (FDA) and the European Medicines Agency (EMA) for the treatment of type 1 SMA, including nusinersen (Spinraza; Biogen), onasemnogene abeparvovec (Zolgensma; Novartis), and risdiplam (Evrysdi; Roche) (16, 17, 33, 36). In our study, the improvement in CHOP-INTEND scores in patients previously treated with nusinersen can be, at least in part, linked to a synergistic mechanism of onasemnogene abeparvovec and nusinersen; however, we also saw substantial improvement in motor function in nusinersen-naïve patients. Although continuation of nusinersen therapy would likely result in improved motor function beyond the 1^st^ year of treatment, and this is also true for risdiplam (37), real-world data in type 1 SMA patients treated with nusinersen show a lower mean change in CHOP-INTEND scores between 12 and 24 months after treatment than that observed from baseline to 12 months (38).

Motor milestones are rarely acquired in untreated type 1 SMA infants, with natural history studies highlighting a failure to achieve major milestones such as rolling over or sitting independently (4, 8, 39, 40). Markedly, all patients in our study achieved motor milestones, with most patients able to sit unassisted for >1 min by the end of the 12-month study period.

Similarly, all patients had higher CHOP-INTEND scores at 12 months. However, when charting the progression of SMA type 1 over time, the CHOP-INTEND is limited by the presence of deformity [such as scoliosis (41)] and tendon retractions, which may affect the results. It is, therefore, imperative that neurocognitive functions are taken into consideration when assessing the global functioning of patients.

Due to their lower life expectancy (9, 42, 43), there has been little consideration of neurocognitive aspects in SMA type 1 patients. In an analysis of SMA type 1–3 patients (aged 6 to almost 19 years), general intelligence was comparable to similar-aged non-affected siblings and healthy controls matched for age, sex, and socioeconomic status (44). However, children with SMA type 1 are more likely to have impaired cognitive processes, including attention and executive function deficits, than children with SMA type II or III (45). Recent studies have also shown that speech quality and motor function are severely affected in untreated SMA type 1 children (46, 47), and that the developmental course of communication from eye fixation to using signs was delayed in SMA type 1 children (48).

To date, few studies have reported the effect of disease-modifying treatments on neurocognitive functioning in children with SMA type 1 (12). One study found that the majority (11 of 12) of infants with SMA type 1 were “able to speak” 2-years after receiving onasemnogene abeparvovec but did not report speech ability at baseline (16, 49). A second study, which used the Griffiths III scale to evaluate the neurocognitive and psychomotor profile of SMA type 1 patients treated with a disease-modifying therapy (mostly nusinersen), reported defects in *Gross Motor* functions but not in the *Foundation of Learning* and *Language and Communication* subscales, suggesting an increase in the development of general neurocognitive abilities (50).

Importantly, our study evaluated the evolution of neurocognitive functioning over a 1-year period after administration of onasemnogene abeparvovec. Like Tosi et al. (50), the *Gross Motor* subscale was the worst preforming subscale irrespective of disease-modifying treatment. Equally, the *Foundations of Learning, Language and Communication, Eye and Hand Coordination*, and *Personal-Social-Emotional* subscale scores increased over time in most patients, except for patient 10 who also had severe cognitive impairment and intellectual disability at baseline.

While untreated SMA type 1 children rarely attain functional and intelligible speech (46, 47), progression in speech and language abilities was observed for most patients in our cohort. Improvements in cognitive performance, language, and communication can partly be explained by the gain in muscle strength and motor performance after gene replacement therapy with onasemnogene abeparvovec. We hypothesize that increased strength and endurance allows greater access to stimulation and cognitive learning thereby increasing the patient's motivation.

Indeed, speech impairment was reported to be strongly correlated with global motor impairment in untreated children with SMA type 1, whereas cognitive development and language comprehension were not correlated with motor function and instead were well preserved (46). With the availability of new therapies, including gene replacement therapy and synthetic antisense oligonucleotides, the natural history of SMA is changing. Consequently, the evaluation and therapeutic-rehabilitative aspects of patients (necessary not only for physiotherapy, but also for psychomotor skills and speech therapy) must also change.

There is a paucity of data on safety and efficacy outcomes of onasemnogene abeparvovec when administered to patients older than 2 years of age. Real-world evidence of 21 patients aged ≥2 years at onasemnogene abeparvovec infusion reported an estimated 3.8-point gain in CHOP-INTEND scores after gene therapy (follow-up duration not reported) (25). A separate study (22) reported an increase of 5.1 points in mean CHOP-INTEND scores from baseline to 12 months (47.1 vs. 52.2 points, respectively) in 19 SMA type 1 patients with ≥2 *SMN2* copies aged >2 years at onasemnogene abeparvovec treatment, which is similar to the 7.7-point increase reported here (46.0 vs. 53.7 points from baseline to 12 months, respectively). Our study also showed new motor milestones, improved language ability, and increased developmental quotient scores in most Griffiths III subscales at 12 months, however, most scores were clinical (< 70) indicating that these patients have an overall developmental delay. Nonetheless, our data suggests that these patients may benefit from gene therapy with onasemnogene abeparvovec in terms of improved motor and neurocognitive functioning despite their advanced age at administration.

Clinical studies have demonstrated a favorable long-term benefit-risk profile of nusinersen in individuals with infantile or late-onset SMA (51). In our patient population, 9 patients had received nusinersen prior to study enrollment, with observed benefits in terms of improvement in motor function and no side effects (20). The decision to switch therapy to onasemnogene abeparvovec was parental and linked to the invasive nature of the nusinersen administration procedure, which requires a lumbar puncture at 0, 14, 28, and 63 days and then every 4 months for life.

Clinical trials and real-world studies of nusinersen, onasemnogene abeparvovec, and risdiplam have demonstrated favorable efficacy and safety data in patients with SMA with two copies of *SMN2* (37, 51, 52). Therefore, the choice of treatment represents a complex process requiring an evaluation of benefits and risks to reach an agreed decision between the family, the referring neurologist, and the multidisciplinary team. Possible side effects must be considered in evaluating the risk–benefit ratio. Notably, treatment with onasemnogene abeparvovec is associated with an increased risk of hepatotoxicity, seen as acute serious liver injury and acute liver failure, including fatal cases, which must be mitigated through adequate monitoring and intervention (13, 53, 54). In our previous study (20), increases in AST and ALT above the normal range were observed primarily in week 1, although the greatest increases were recorded in week 5. However, elevated transaminases were successfully managed with corticosteroids in all cases.

Of course, our study is limited by low patient numbers, the use of a non-standardized neurodevelopmental test for SMA, the omission of a control group, and a lack of data on the intellectual development of the treated SMA type 1 patients. In addition, nine patients were pretreated with nusinersen, which has a long half-life, hence it cannot be certain that the effects seen were related to onasemnogene abeparvovec alone. Nonetheless, positive outcomes after onasemnogene abeparvovec were observed in all nusinersen-naïve patients supporting its efficacy in both motor and neurocognitive outcomes.

An important finding of our study is that treated symptomatic SMA type 1 patients with two copies of *SMN2* have impairments beyond motor function that include all domains in the Griffiths Scale. While it remains unclear as to what extent there is interdependency between motor function and evaluated Griffiths subscale scores, the longitudinal data suggest that neurocognitive scores as measured by Griffiths are increasing over 1 year. However, attributing this change to disease modifying therapies is tenuous without controls or an alternative study design.

## 5 Conclusion

Our results add to the growing evidence supporting the efficacy and safety of onasemnogene abeparvovec in patients with SMA type 1. Although the study population was small, which limits our ability to definitively define the impact of onasemnogene abeparvovec on neurocognitive functioning, most patients showed some improvement in cognitive, verbal, and communication skills alongside motor functioning and motor milestone achievements. As the aim of our study was to underline the importance of cognitive evaluation in SMA type 1 children both at diagnosis and during clinical evolution, these outcomes reinforce the importance of considering neurocognitive aspects when assessing the global functioning of patients with SMA type 1.

Our study also shows that the current standardized scales used to assess patients with SMA type 1 (such as the CHOP-INTEND) measure only gross motor skills. As these scales do not assess fine motor skills, acquired autonomy, communication, or cognitive skills, they do not accurately reflect a patient's global functioning. Hence, additional age-appropriate scales for fine motor skills and other child developmental domains should be introduced. With the new therapies and changes in the clinical evolution of SMA, it would now be appropriate to devise new standardized evaluation scales that consider the numerous aspects of neurodevelopment.

## Data availability statement

The raw data supporting the conclusions of this article will be made available by the authors, without undue reservation.

## Ethics statement

The studies involving humans were approved by AORN Santobono-Pausilipon Ethics Committee. The studies were conducted in accordance with the local legislation and institutional requirements. Written informed consent for participation in this study was provided by the participants' legal guardians/next of kin. Written informed consent was obtained from the individual(s) and minor(s)' legal guardian/next of kin, for the publication of any potentially identifiable images or data included in this article.

## Author contributions

IB: Conceptualization, Data curation, Investigation, Methodology, Project administration, Writing—original draft, Writing—review & editing. MM: Data curation, Investigation, Writing—review & editing. RS: Data curation, Investigation, Writing—review & editing. AV: Conceptualization, Data curation, Investigation, Project administration, Supervision, Writing—review & editing.
